# The Appropriateness of Empiric Treatment of Urinary Tract Infections in a Tertiary Teaching Hospital in Joran: A Cross-Sectional Study

**DOI:** 10.3390/antibiotics11050629

**Published:** 2022-05-06

**Authors:** Rama Alkhawaldeh, Rana Abu Farha, Khawla Abu Hammour, Eman Alefishat

**Affiliations:** 1 Department of Clinical Pharmacy, Faculty of Pharmacy, Applied Science Private University, Amman 11931, Jordan; ramabaker73@gmail.com (R.A.); r_abufarha@asu.edu.jo (R.A.F.); 2 Department Biopharmaceutics and Clinical Pharmacy, Faculty of Pharmacy, The University of Jordan, Amman 11942, Jordan; k.hammour@ju.edu.jo; 3 Department of Pharmacology, College of Medicine and Health Science, Khalifa University of Science and Technology, Abu Dhabi 127788, United Arab Emirates; 4 Center for Biotechnology, Khalifa University of Science and Technology, Abu Dhabi 127788, United Arab Emirates

**Keywords:** antibiotic resistance, empiric antibiotic, urine culture, susceptibility test, Jordan

## Abstract

This is a cross-sectional study that was conducted at Jordan University Hospital (JUH) to evaluate the appropriateness of Urinary Tract Infection (UTI) empiric treatment based on microbial culture data and susceptibility testing. All urine cultures requested for adult patients (≥18 years) admitted to JUH within the period from January 2019–July 2021 were reviewed and only those cultures with positive episodes of infection were considered. In this study, 6950 urine culture episodes were screened; among them, 34.5% (*n* = 2400) revealed positive results. Among those patients with positive culture episodes, 1600 patients (66.7%) were discharged before the availability of culture results and were excluded. Of the remaining eligible 800 patients, 701 (87.6%) received empiric treatment. In 26.8% of the eligible cases (*n* = 214), the prescribed empiric agents failed to have appropriate coverage of the identified pathogens, and in 14.6% of the cases (*n* = 117) the identified microorganisms were reported as resistant to the prescribed empiric agents. Furthermore, only 13.4% of the patients (*n* = 107) were appropriately treated for their UTI with empiric antibacterial agents. We were not able to judge the appropriateness of UTI treatment for one third (*n* = 263, 32.9%) of the patients, because they did not have susceptibility reports performed. This study revealed an alarmingly high rate of inappropriate treatment of UTIs, which encourages the emergence of bacterial resistance and affects health-related outcomes negatively. Therefore, antimicrobial stewardship programs must be applied to optimize antibiotic consumption in hospital settings.

## 1. Introduction

Antimicrobial Resistance (AMR) is the ability of microorganisms, including bacterial pathogens, to survive and grow despite exposure to substances that are supposed to kill them [1]. AMR has become one of the fastest-growing threats in global health and it is associated with increased morbidity and mortality, in addition to disastrous economic impacts due to treatment failure and prolonged hospitalization [2,3]. Several factors have been reported to increase the risk of bacterial resistance, including the widespread use of antibacterial agents in humans as well as in animals [4]. Increased prescription rates of antibiotics by physicians may occur because medication choice is based on both cost and toxicity, where the antibiotic with the lowest cost and toxicity is chosen [5].

Urinary tract infections (UTIs) are frequent infections in hospitalized and non-hospitalized patients of all ages and both genders, although more common in females [6]. There is a documented need to address UTIs as soon as possible to avoid serious possible complications [7,8]. However, it is judicious to limit the use of antibiotics to appropriate cases in order to curb antibiotic resistance and reduce the risk of adverse drug reactions [9]. It can be easily supposed that if we optimize antibacterial use this will lead to a reduction in resistance [10].

Several treatment options are available to manage the different classes and severities of UTIs and their symptoms [11]. According to multiple studies, carbapenems, fluoroquinolones, trimethoprim–sulfamethoxazole, aminoglycosides, and beta-lactam antibiotics are the most extensively utilized broad spectrum antibacterial agents [11]. To have an optimal treatment for UTIs, physicians should prescribe an appropriate empiric antibiotic that covers the identified microorganisms with the confirmation of the uropathogen’s susceptibility to that empiric treatment [12]. Previous studies have shown the value of culture and sensitivity testing in decreasing the inappropriate antibacterial use [13,14]. After obtaining antibiotic susceptibility results and microbial culture data, the antibiotic regimen should be re-evaluated based on the patient’s current condition [15]. First, physicians should review the body site from which the sample was isolated and showed a positive result [15]. Second, the results of susceptibility tests provide an opportunity for treatment, ranging from a broad-spectrum antibiotic to targeted therapy with a narrow-spectrum antibiotic in order to decrease unnecessary antibacterial exposure and increase the antibiotic’s effectiveness [15].

The purpose of this study was to evaluate the appropriateness of treatment of UTI patients based on microbial culture data and susceptibility results in a tertiary teaching hospital in Jordan. 

## 2. Methods

### 2.1. Study Design and Participants, and Clinical Setting

This was a cross-sectional study that was conducted at Jordan University hospital, which is the first academic teaching hospital in Jordan with a 550-bed capacity. This study reviewed all urine cultures requested for patients (≥18 years) admitted to JUH within the last two years (January 2019–July 2021), and only those cultures with positive episodes of infection were considered. To avoid replication of patients with multiple urine cultures on the same admission, data were only collected from the first culture per admission. In addition, data regarding antibiotic susceptibility testing was also collected (if available) for each included patient.

Following the gathering of information on culture and susceptibility testing, medical files were reviewed for any antibiotics prescription within five days before obtaining culture results, and only patients with documented antibiotic prescriptions were considered for the evaluation of the appropriateness of UTI treatment. Patients with no antibiotic prescription within the recommended time window were considered to have untreated conditions. Other information collected included each patient’s gender, age, length of hospitalization, and number of chronic medications.

### 2.2. Main Data Sources

Information regarding urine culture and susceptibility testing were obtained from the JUH laboratory’s electronic system, together with details of the prescribed empiric antibiotics and other medical records for each patient.

### 2.3. Evaluation of the Appropriateness of the of Urinary Tract Infection Empiric Treatment

Urinary tract infections are considered to be appropriately treated with empiric antibiotic if that antibiotic is prescribed prior to urine culture results, and if the identified microorganism(s) is/are within the spectrum covered by the empiric antibiotic [16]. If so, the same empiric antibiotic was evaluated for its sensitivity based on the susceptibility testing. Treatment with antibiotic was considered as appropriate if the bacteria were reported as susceptible to that antibiotic. In order for the treatment of the UTI to be considered appropriate, the empiric antibiotic should have appropriate selection and the microorganism should be susceptible to it.

### 2.4. Ethical Consideration

The World Medical Association Declaration of Helsinki guidance was followed in the study [17], which was initiated after obtaining approval by the Institutional Review Board (IRB) committee at Jordan University Hospital (Reference No. 196/2021). All the collected information was kept on the personal computer of the principal investigator using password-protected files.

### 2.5. Statistical Analysis

All the collected data were coded, entered, and analyzed using the Statistical Package for Social Sciences (SPSS) version 22. The descriptive analysis was conducted using mean and standard deviation (SD) for continuous variables and percentages for categorical variables.

## 3. Results

### 3.1. Demographic and Medical Characteristics of the Study Sample

During the study period, 6950 urine culture episodes from the same number of patients were screened and among them 65.5% (*n* = 4550) were negative culture episodes that were excluded from the study. The remaining 34.5% (*n* = 2400) revealed positive results. Among those patients with positive culture episodes, 1600 patients (66.7%) were discharged too early, before the availability of culture results, and were also excluded.

The median age of the remaining eligible patients (*n* = 800) was 64 years (IQR = 29), with 71.0% of the participants (*n* = 568) above 50 years old, and more than two thirds of them females (*n* = 555, 69.4%). Moreover, more than half of the patients (*n* = 437, 54.6%) were receiving polypharmacy (≥ 4 medications) and they had a median length of stay of 12 days (IQR = 11). For more details about the demographic and medical characteristics of the study sample, refer to Table 1.

### 3.2. Empiric Antibiotic Prescribing

**Figure 1:**
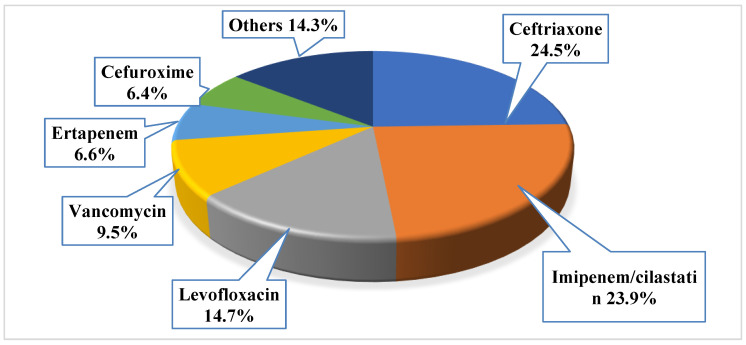
The prescribed empiric antibacterial among the study sample (note: the total number of prescribed empiric antibiotics was 873).

Antibiotics were primarily prescribed before the culture result as empirical prescription for 701 patients (87.6%). The mean number of the prescribed empiric antibiotics for those with positive urine culture was 1.1 ± 0.6, with a total of 873 prescribed empiric antibacterial agents. Around 12% of the patients (*n* = 99, 12.4%) did not receive any antibacterial agent, while the majority had received one antibacterial agent (*n* = 556, 69.5%), 14.8% of them had received two antibacterial agents (*n* = 118), and only 3.4% of them had received three antibacterial agents (*n* = 27). The most frequently prescribed empiric antibiotics were ceftriaxone (*n* = 214, 24.5%), imipenem/cilastatin (*n* = 209, 23.9%), and levofloxacin (*n* = 128, 14.7%); the least prescribed agents were tigacyclin, cefotaxime, ampicillin, and linezolid (*n* = 1, 0.1% for each). More details about the most frequently prescribed empiric antibiotics, refer to Figure 1.

### 3.3. Urine Culture Testing and Susceptibility Testing

**Figure 2:**
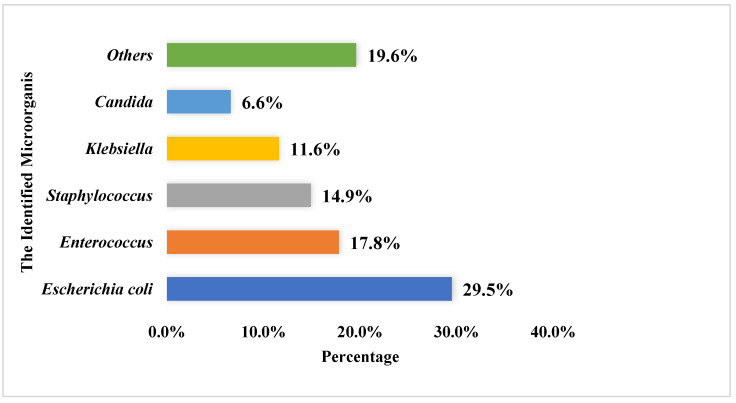
The most commonly identified microorganisms within the urine culture among the study sample (the total number of identified microorganism was 1062).

Most urine culture specimens revealed one microorganism (*n* = 559, 69.9%), with only a few specimens showing two or more pathogens (241, 30.1%). The mean number of pathogens listed in culture reports was 1.3 ± 0.5, and the most frequently reported pathogens among the urine cultures were *E. coli* (*n* = 313, 29.5%), *Enterococcus* (*n* = 189, 17.8%), and *Staphylococcus* (*n* = 158, 14.9%). For more details, refer to Figure 2.

Among the study sample, around half of the patients (*n* = 391, 48.9%) had susceptibility report performed. The identified microorganisms were reported as resistant to 136 out of the 873 prescribed antibiotics (15.6%). These antibiotics reported as “resistant” were prescribed for 117 out of the 800 eligible patients (14.6%). The most commonly prescribed antibiotics that have acquired the greatest resistance from bacteria were ceftriaxone (*n* = 48, 35.3%), levofloxacin (*n* = 27, 19.9%), and imipenem/cilastatin (*n* = 21, 15.4%). Details of susceptibility results are presented in Table 2.

### 3.4. Evaluation of the Appropriateness of Urinary Tract Infection Treatment

**Figure 3:**
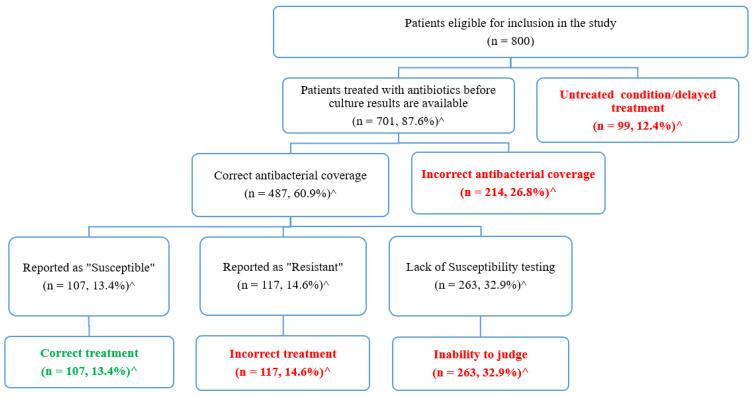
Evaluation of the appropriateness of urinary tract infection treatment based on the culture and susceptibility results. ^ Percentages were calculated based on the total number of eligible patients (*n* = 800).

The appropriateness of urinary tract infection treatments was evaluated based on the urine culture and susceptibility data (Figure 3). As seen in Figure 3, 12.4% of the eligible patients (*n* = 99) received no antibiotics before culture results, and they were considered to have untreated conditions or delayed treatments, whereas the remaining 701 (87.6%) patients received antibacterial treatments. Each antibacterial agent was evaluated to assess if its spectrum of activity covered the identified microorganisms. Accordingly, in 214 patients (26.8%) the prescribed antibiotics failed to have the appropriate coverage of the identified pathogens. The remaining 487 patients (60.9%) were treated with agents that cover the identified pathogens.

Subsequently, susceptibility tests were used to evaluate whether the identified pathogens were susceptible to the prescribed agents. As shown in Figure 3, susceptibility tests were not available for 263 patients (32.9%), so we were not able to judge the appropriateness of the prescribed agents among those patients. The remaining patients were divided into two groups: those who were prescribed incorrect agents that were reported as “resistant” (*n* = 117, 14.6%), and those who were correctly treated with agents that were reported as “sensitive” (*n* = 107, 13.4%).

Overall, among the 800 eligible patients only 13.4% were appropriately treated with empiric antibacterial agents (*n* = 107), while the remaining 693 (86.6%) were inappropriately treated or left untreated. Table 3 presents several examples of the different types of inappropriate treatment with empiric antibacterial agents.

## 4. Discussion

This study evaluated the appropriateness of UTI empiric treatment for 800 adult hospitalized patients, based on microbial culture data and susceptibility. Among those eligible patients, 12.4% received no empiric treatment, while in 26.8% of the cases the prescribed empiric agents failed to have appropriate coverage of the identified pathogens, and in 14.6% of the cases the identified microorganisms were reported as resistant to the prescribed empiric agents. Furthermore, only 13.4% of the patients were appropriately treated for their UTI with empiric antibacterial agents. Moreover, we were not able to judge the appropriateness of UTI treatment for one third (32.9%) of the patients because they did not have susceptibility reports performed, revealing an alarmingly high rate of inappropriate treatment of UTIs.

Among patients with available culture results prior to their discharge, 12.4% of them did not receive any empiric antibacterial agents before the culture results were available, which could worsen their conditions. A similar finding was reported in a study conducted by Zhu et al. at a tertiary hospital in southern China, where 15.7% of the patients with positive urine did not receive empiric treatment [18]. This delay in antibiotic administration may negatively affect the outcome of the disease and increase the economic burden, as well as having a greater influence on patient outcomes than the antibiotics resistance itself [19].

The remaining percentage of our study sample (87.6%) received empiric treatment. Our results show that 26.8% of the prescribed empiric agents failed to have the appropriate coverage of the identified pathogens, which negatively affected the health-related outcome in these patients. According to a recent study conducted in Ireland, the insufficient management of infections was associated with increased morbidity and death, while excessive or inappropriate antibacterial use contributed to the emergence of the antibiotic resistance [20].

The purpose of sensitivity testing is to identify probable drug resistance in common organisms and to ensure sensitivity to certain antibiotics [21]. In this study, 14.6% of patients were found to be incorrectly treated empirically for their UTIs, since their identified microorganisms were found to be resistant to the empiric agents they received, despite the correct coverage. Moreover, only 13.4% of the study sample were appropriately treated by receiving agents that were reported as “sensitive”. Similar findings were reported in a study conducted in South Sudan, where only 21% of patients were found to have been prescribed the appropriate antibiotic therapy [22]. Another study found a higher estimated annual appropriate antibiotic prescription rate of 353 out of 506 antibiotic prescriptions per 1000 population [23].

Unfortunately, and against international recommendations, microbiological susceptibility tests were not carried out for many of the studied patients, so we were not able to report on the appropriateness of the prescribed empiric antibacterial agents in one third of participating patients (32.9%) since they had no sensitivity testing. This percentage was close to that reported by Tellado et al. where 46.8% of the patients did not have sensitivity testing [24].

The overall level of the inappropriate management of UTIs in this study was high (86.6%). The forms of inappropriate management were either in the form of insufficient treatment of the condition, inappropriate coverage of the antibiotics, the resistance of the identified microorganism to empiric therapy, or the lack of sensitivity testing. This large percentage of inappropriate management of UTIs among study participants is substantial and necessitates immediate attention as they are significantly higher than those reported in other parts of the world. According to a recent study conducted in Spain, only 9 % of patients received improper empiric antibacterial medication [24], while a study by Tünger et al. at Celal Bayar university hospital in Turkey reported that incorrect empiric antibiotic use was found in 54.3% of patients [25].

In our study, the majority of patients (69.5%) had only received one empiric antibacterial medication. This is higher than the rates reported in a study conducted in Spain, which reported that 45.2% of patients received a single antibiotic, and 48.9% received two antibiotics [24]. However, our results are similar to those reported by Tünger et al., performed where most patients received only one empiric antibiotic. In addition, they concluded that the appropriate usage of antibiotics in patients who received mono empirical antibacterial therapy was 49.4% [25]. In a study that compared cefepime as a mono-empiric antibacterial agent with a combination of broad-spectrum antibiotics, it was found that cefepime was similar to combinational therapy in terms of efficacy [26]. Another study was conducted in Canada to compare the efficacy and outcome of meropenem and ciprofloxacin as empiric therapy versus meropenem alone as monotherapy. They concluded in their randomized clinical trial that meropenem monotherapy contributed to the same efficacy and outcome as combinational therapy [27].

The results of our study show that ceftriaxone was the most commonly prescribed empiric antibacterial agent (24.5%), and the predominant number of pathogens reported were Gram-negative bacilli. *E. coli* was the most frequently reported pathogen according to urine culture testing, followed by *Enterococcus* and *Staphylococcus* (29.5%, 17.8%, and 14.9%), respectively. This agreed with previous findings in the literature, which indicated that *E. coli* was the most commonly identified Gram-negative bacteria in both hospitalized and outpatient individuals with UTIs, and that ceftriaxone was one of the most commonly prescribed antibacterial agents in empiric treatment [1]. However, a study in Singapore aimed to identify if there was an association between the extensive use of empiric ceftriaxone and the potential for resistance to develop; they concluded that ceftriaxone resistance was found in 33.3% of the isolated *E. coli* species [28]. A similarly high rate of resistance was reported in our study for ceftriaxone (35.3%). These results support what was previously proposed, namely that the excessive use of antibacterial agents promotes and accelerates the emergence of bacterial resistance [28].

Finally, this study has some limitations. Firstly, the patients’ data were obtained from the database of a single tertiary center in Jordan (JUH), which cannot represent patients nationwide and the results might not be generalizable. However, as the first study of its kind in Jordan, and bearing in mind the observational type of the study, it is considered satisfactory to provide background data at this stage. Secondly, the study revealed that an alarmingly high percentage of patients (66.7%) with a positive urine culture were excluded since they were discharged too early, before the availability of culture results. Thirdly, the evaluation of the appropriateness of the UTI empiric treatment was judged based on the empirical treatment and the diagnosis only, without knowing the history of the patient or what drove the physician to prescribe a certain drug. Moreover, without knowing patient medical history, it was not possible to evaluate the quality of the prescription according to the clinical practice guidelines.

## 5. Conclusions

This study revealed an alarmingly high rate of inappropriate prescribing of empiric antibiotics for treating UTIs, which encourages the emergence of bacterial resistance and results in a deterioration in a patient’s outcome. Therefore, antimicrobial stewardship programs must be applied to optimize antibiotic consumption in hospital settings. Moreover, standard treatment guidelines must be followed to reduce the irrational use of antibiotics and the probability of developing antibiotic resistance.

## Figures and Tables

**Table 1:** Demographic and medical characteristics of the study sample (*n* = 800).

Parameter	Results
Age in years, Median (IQR)	64.0 (29.0)
Age categories (years), *n* (%)	
o 20–50.0	232 (29.0)
o 50.1–80.0	483 (60.4)
o 80.1–110	85 (10.6)
Gender, *n* (%)	
o Female	555 (69.4)
o Male	245 (30.6)
Number of chronic medications, *n* (%)	
o 0–1	178 (22.3)
o 2–3	185 (23.1)
o ≥4	437 (54.6)
Length of Stay, Median (IQR)	12.0 (11.0)

IQR: interquartile range.

**Table 2:** Details of susceptibility results among the study sample.

Parameter	*n* (%)
The availability of susceptibility reporting#	
o No	409 (51.1)
o Yes	391 (48.8)
Number of patients with resistant microorganism#	117 (14.6)
The most commonly prescribed antibiotics that have acquired the greatest resistance from bacteria ^	
o Ceftriaxone	48 (35.3)
o Levofloxacin	27 (19.9)
o Imipenem/Cilastatin	21 (15.4)
o Cefuroxime	14 (10.3)
o Pipercillin/Tazobactam	7 (5.1)
o Others	19 (14.0)
o Total	136

# Percentage calculated from the total number of eligible patients (*n* = 800). ^ Percentage calculated from the total number of antibiotics reported as resistance (*n* = 136).

**Table 3:** Examples of different types of the inappropriate empiric treatment of urinary tract infection.

Inappropriateness of UTI Empiric Treatment	
Untreated condition	A 72-year-old male admitted to the JUH. The results of urine culture showed the presence of *Klebsiella and Staphylococcus*. The patient did not receive any intravenous antibiotics before the results of culture were available.
Incorrect antibacterial coverage	A-29-year-old female admitted to the JUH. The results of urine culture showed the presence of *Pseudomonas aeruginosa*. The patient received Cefazolin as an empiric antibiotic before the results of culture were available. The empiric antibiotic was not correct because of the lack of coverage.
Incorrect treatment (The identified pathogens were reported as resistant to the prescribed agents)	A 60-year-old female admitted to the JUH. The results of urine culture showed the presence of *E. coli*. The patient was given Ceftriaxone as an empiric antibiotic before results of culture were available. The empiric antibiotic was not correct because the identified pathogen was reported to be resistant to Ceftriaxone.
Inability to judge (Lack of susceptibility testing) Not performed at all o Not performed for this antibiotic	A 76-year-old male admitted to the JUH. The results of urine culture showed the presence of *E. coli*. Meropenem was given to the patient as an empiric antibiotic before results of culture were available. We could not conclude the appropriateness of the use of Meropenem since no sensitivity test was performed. A 67-year-old male admitted to the JUH. The results of urine culture showed the presence of *Acinetobacter*. Colistin was given to the patient as an empiric antibiotic before results of the culture were available. We could not conclude the appropriateness of the use of Colistin since no sensitivity test was performed for this particular antibiotic.
Correct treatment (The identified pathogens were reported as susceptible to the prescribed agent)	An 87-year-old female admitted to the JUH. The urine culture results showed the presence of *Staphylococcus*. Vancomycin was given to the patient as an empiric antibiotic before the culture results were available. Depending on the results of the sensitivity test, the use of this antibiotic is considered appropriate.

## Data Availability

The data presented in this study is available in the article.

## References

[1] Harbottle H., Thakur S., Zhao S., White D. (2006). Genetics of antimicrobial resistance. Anim. Biotechnol..

[2] Tebano G., Mouelhi Y., Zanichelli V., Charmillon A., Fougnot S., Lozniewski A., Thilly N., Pulcini C. (2020). Selective reporting of antibiotic susceptibility testing results: A promising antibiotic stewardship tool. Expert Rev. Anti-Infect. Ther..

[3] Davison H.C., Woolhouse M.E., Low J.C. (2000). What is antibiotic resistance and how can we measure it?. Trends Microbiol..

[4] Byarugaba D. (2004). Antimicrobial resistance in developing countries and responsible risk factors. Int. J. Antimicrob. Agents.

[5] Griffith M., Postelnick M., Scheetz M. (2012). Antimicrobial stewardship programs: Methods of operation and suggested outcomes. Expert Rev. Anti-Infect. Ther..

[6] Karlowsky J.A., Kelly L.J., Thornsberry C., Jones M.E., Sahm D.F. (2002). Trends in antimicrobial resistance among urinary tract infection isolates of Escherichia coli from female outpatients in the United States. Antimicrob. Agents Chemother..

[7] Karavanaki K.A., Soldatou A., Koufadaki A.M., Tsentidis C., Haliotis F.A., Stefanidis C.J. (2017). Delayed treatment of the first febrile urinary tract infection in early childhood increased the risk of renal scarring. Acta Paediatr..

[8] Johnson J.R., Russo T.A. (2018). Acute Pyelonephritis in Adults. N. Engl. J. Med..

[9] Lee C.-R., Cho I.H., Jeong B.C., Lee S.H. (2013). Strategies to minimize antibiotic resistance. Int. J. Environ. Res. Public Health.

[10] Reygaert W.C. (2018). An overview of the antimicrobial resistance mechanisms of bacteria. AIMS Microbiol..

[11] Carson C., Naber K.G. (2004). Role of fluoroquinolones in the treatment of serious bacterial urinary tract infections. Drugs.

[12] Murthy R. (2001). Implementation of strategies to control antimicrobial resistance. Chest.

[13] Tabak Y.P., Vankeepuram L., Ye G., Jeffers K., Gupta V., Murray P.R. (2018). Blood culture turnaround time in US acute care hospitals and implications for laboratory process optimization. J. Clin. Microbiol..

[14] Maugeri G., Lychko I., Sobral R., Roque A.C. (2019). Identification and antibiotic-susceptibility profiling of infectious bacteria. Biotechnol. J..

[15] LaPlante K., Cunha C., Morrill H., Rice L., Mylonakis E. (2016). Antimicrobial Stewardship: Principles and Practice.

[16] Harvey R.A., Clark M., Finkel R., Rey J., Whalen K. (2012). Lippincott’s Illustrated Reviews: Pharmacology.

[17] World Medical A. (2013). World medical association declaration of helsinki: Ethical principles for medical research involving human subjects. JAMA.

[18] Zhu H., Chen Y., Hang Y., Luo H., Fang X., Xiao Y., Cao X., Zou S., Hu X., Hu L. (2021). Impact of inappropriate empirical antibiotic treatment on clinical outcomes of urinary tract infections caused by Escherichia coli: A retrospective cohort study. J. Glob. Antimicrob. Resist..

[19] Lodise T.P., Berger A., Altincatal A., Wang R., Bhagnani T., Gillard P., Bonine N.G. (2019). Antimicrobial resistance or delayed appropriate therapy—Does one influence outcomes more than the other among patients with serious infections due to carbapenem-resistant versus carbapenem-susceptible Enterobacteriaceae?. Open Forum Infect. Dis..

[20] O’Grady M.C., Barry L., Corcoran G.D., Hooton C., Sleator R.D., Lucey B. (2019). Empirical treatment of urinary tract infections: How rational are our guidelines?. J. Antimicrob. Chemother..

[21] Reller L.B., Weinstein M., Jorgensen J.H., Ferraro M.J. (2009). Antimicrobial susceptibility testing: A review of general principles and contemporary practices. Clin. Infect. Dis..

[22] Alharafsheh A., Alsheikh M., Ali S., Baraiki A., Alharbi G., Alhabshi T., Aboutaleb A. (2018). A retrospective cross-sectional study of antibiotics prescribing patterns in admitted patients at a tertiary care setting in the KSA. Int. J. Health Sci..

[23] Fleming-Dutra K.E., Hersh A.L., Shapiro D.J., Bartoces M., Enns E.A., File T.M., Finkelstein J.A., Gerber J.S., Hyun D.Y., Linder J.A. (2016). Prevalence of Inappropriate Antibiotic Prescriptions Among US Ambulatory Care Visits, 2010–2011. JAMA.

[24] Tellado J.M., Sen S.S., Caloto M.T., Kumar R.N., Nocea G. (2007). Consequences of inappropriate initial empiric parenteral antibiotic therapy among patients with community-acquired intra-abdominal infections in Spain. Scand. J. Infect. Dis..

[25] Tünger Ö., Dinç G., Özbakkaloglu B., Atman Ü.C., Algün Ü. (2000). Evaluation of rational antibiotic use. Int. J. Antimicrob. Agents.

[26] Badaró R., Molinar F., Seas C., Stamboulian D., Mendonça J., Massud J., Nascimento L.O. (2002). A multicenter comparative study of cefepime versus broad-spectrum antibacterial therapy in moderate and severe bacterial infections. Braz. J. Infect. Dis..

[27] Heyland D.K., Dodek P., Muscedere J., Day A., Cook D., Canadian Critical Care Trials Group (2008). Randomized trial of combination versus monotherapy for the empiric treatment of suspected ventilator-associated pneumonia. Crit. Care Med..

[28] Li N.Y., Poh G.Q., Teng G.C.W., Chen H.H., Chan D.S.G., Chan S.-P., Tambyah P.A., Bagdasarian N., Wu J.E. (2020). A prediction tool for the presence of ceftriaxone-resistant uropathogens upon hospital admission. Antibiotics.

